# Microbial valorization of underutilized and nonconventional waste streams

**DOI:** 10.1093/jimb/kuab056

**Published:** 2021-09-16

**Authors:** Beena C Lad, Sarah M Coleman, Hal S Alper

**Affiliations:** Department of Molecular Biosciences, The University of Texas at Austin, 100 East 24th St. Stop A5000, Austin, Texas 78712, USA; McKetta Department of Chemical Engineering, The University of Texas at Austin, 200 E. Dean Keeton St. Stop C0400, Austin, Texas 78712, USA; McKetta Department of Chemical Engineering, The University of Texas at Austin, 200 E. Dean Keeton St. Stop C0400, Austin, Texas 78712, USA; Institute for Cellular and Molecular Biology, The University of Texas at Austin, 2500 Speedway Avenue, Austin, Texas 78712, USA

**Keywords:** Microbial valorization, Pretreatment, Inhibitors, Industrial waste

## Abstract

The growing burden of waste disposal coupled with natural resource scarcity has renewed interest in the remediation, valorization, and/or repurposing of waste. Traditional approaches such as composting, anaerobic digestion, use in fertilizers or animal feed, or incineration for energy production extract very little value out of these waste streams. In contrast, waste valorization into fuels and other biochemicals via microbial fermentation is an area of growing interest. In this review, we discuss microbial valorization of nonconventional, aqueous waste streams such as food processing effluents, wastewater streams, and other industrial wastes. We categorize these waste streams as carbohydrate-rich food wastes, lipid-rich wastes, and other industrial wastes. Recent advances in microbial valorization of these nonconventional waste streams are highlighted, along with a discussion of the specific challenges and opportunities associated with impurities, nitrogen content, toxicity, and low productivity.

## Introduction

A sustainable, circular economy eliminates waste through dematerialization and reuse of material, especially carbon. Although this sustainability paradigm first emerged in the 1960s, it is becoming increasingly relevant (and fortunately, even more technologically feasible) in recent years in light of population and economic growth as well as global industrialization. The many consequences of our single-use economy—from climate change, pollution, land and resource scarcity, and biodiversity loss—necessitate a shift in societal perspective on waste. As a result of this line of thinking, waste streams are increasingly being recognized as valuable. For example, agricultural waste streams rich in lignocellulosic biomass such as corn stover, rice and wheat straw, and sugarcane bagasse have been explored for biofuels and specialty chemicals production (Singhvi & Gokhale, 2019). Wastewater treatment plant and paper mill sludge have likewise received a great deal of interest (Brown et al., 2021; Du et al., 2020; Ouadi et al., 2019; Vashistha et al., 2019) as unique carbon sources. Aside from these streams, most industrial and agricultural wastes have received little attention. Some of these wastes have been explored as fertilizers or animal feed, although this is limited by the presence of antinutritional factors and toxicants (Dou et al., 2018; Mo et al., 2018; Zhang et al., 2020). Across the board, valorization approaches (both biological and chemical) are required to convert these various waste streams into valuable fuels and chemicals.

Perhaps the most ubiquitously evaluated microbial technology for biological waste remediation is anaerobic digestion (AD), a process that produces biogas (primarily a mixture of methane and CO_2_) typically used for lighting and heating, and digestate, typically applied to land as a fertilizer. This microbial technology is not easily scalable and extracts minimal value from waste. Moreover, biogas is not feasible for many energy uses due to poor quantity and quality of methane and presence of impurities (especially H_2_S). Further upscaling of this biogas directly produces biomethane, which may be compressed to create a transportation fuel (compressed natural gas) or reformed to produce syngas (Nguyen et al., 2019; Rasapoor et al., 2020). The other half of the AD products, the digestate, must also undergo treatment prior to use to prevent public health risks (Surendra et al., 2014). As a result of these limitations, new approaches are needed to truly valorize these waste streams.

**Fig. 1:**
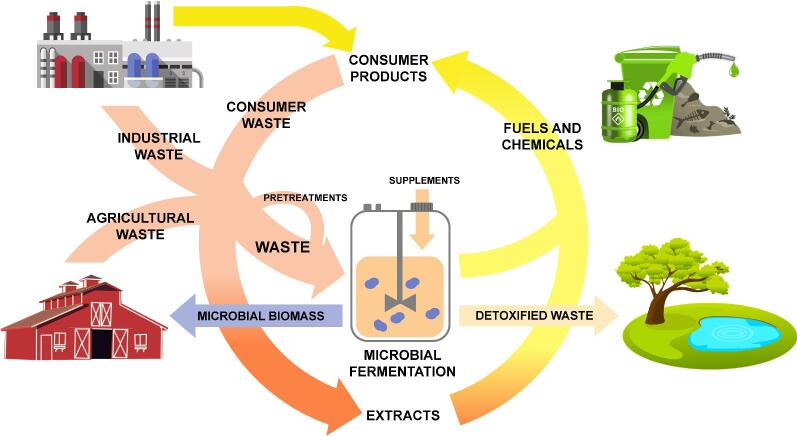
*Graphical depiction illustrating the role of microbial fermentation in a circular economy*. Microbial fermentation valorizes resources back toward high-value products, fuels, and platform chemicals while detoxifying waste and generating biomass, with side stream applications such as fertilizer or animal feed. Images are obtained from open source resources including Pixabay and Openclipart.

Thermochemical and electrochemical conversion technologies have been explored as alternatives to upgrade inorganic wastes (Yoldi et al., 2019) and industrial streams such as saline waste (Baena-Moreno et al., 2020) and purified glycerol (Aroua & Cognet, 2020; Lin, 2013). However, most industrial wastes are complex mixtures that are better treated using microbial fermentation as a valorization approach. More specifically, microorganisms are able to metabolize structurally distinct compounds at mild conditions and route them toward a single product, a feat incredibly difficult using purely chemical technologies. Host selection can be achieved by selecting either native isolates from waste streams (Bhatt et al., 2019) or through metabolically engineered hosts using enzymes from these isolates. Regardless of the tact, microbial fermentation reroutes carbon toward high-value products while detoxifying waste, as depicted in Fig. 1, and will therefore be a critical technology for waste valorization and achieving a circular economy.

Microbial platforms have been established to produce high-value products such as pharmaceuticals, surfactants, platform chemicals, and biodegradable polymers from industrial streams. In addition to extracting value, microbial valorization also reduces the treatment costs traditionally associated with waste disposal. Recent advances in synthetic biology and metabolic engineering are expanding both the breadth of available waste streams and the uniqueness of downstream products. In this review, we highlight recently developed microbial fermentation technologies for valorizing underutilized wastes, and discuss the challenges associated with impurities, nitrogen content, toxicity, and low productivity. Select examples illustrating strategies to overcome these challenges are highlighted in Table 1. In particular, we discuss nonconventional, aqueous waste streams originating from food-processing effluents, wastewater streams, and other industrial wastes. These waste streams are broadly categorized as carbohydrate-rich food wastes, lipid-rich wastes, and other industrial wastes. Throughout, we focus on the potential titers as well as the metabolic engineering strategies required to achieve valorization.

**Table 1.** Representative Examples of Challenges in Microbial Valorization of Various Waste Streams and Recent Advances to Overcome Them

Valorization challenge—feedstock	Remediation strategy	Product	Titer, Yield	Scale	Organism	Fermentation mode	Description or notes	Reference
Impurities—microbial inhibitors in fruit peels	Distillation to extract limonene and plant essential oils	Succinic acid	22.4 g/l, 0.73 g/g	0.45 l fed-batch bioreactor, 0.3 l working volume	*Actinobacillus succinogenes* Z130	Aerobic	Essential oils (vol/wt): 0.43% ground Mandora peel, 0.19% nonground Mandora peel, 0.24% household kitchen citrus waste, mainly D-limonene	Patsalou et al. (2020)
		Bioflocculants	3.49 g/l	Not specified	*Alcaligenes faecalis* subsp*. phenolicus* ZY-16	Aerobic	Limonene removal improved titer from 1.06–3.04 g/l, medium optimization afterward yielded further improvement, citrus waste	Qi et al. (2020)
	Selection of tolerant host strain	Lipids	1.68 g/l (0.055 g/g) on undetoxified hydrolysate, 1.83 g/l (0.057 g/g) on detoxified	500 ml flask, 150 ml working volume	*Rhodotorula mucilaginosa* KKUSY14	Aerobic	Durian peel hydrolysate	Siwina and Leesing (2021)
Incomplete consumption—host cannot consume all sugars in citrus peel hydrolysate	Engineer D-galacturonic acid metabolism, Expression of fungal GatA transporter	Meso-galactaric acid	8.0 ± 0.6 g/l with glucose supplementation, 3.2 ± 0.1 g/l without supplementation	250 ml baffled flask	*Saccharomyces cerevisiae* BY4741	Aerobic	Also improved redox balance	Protzko et al. (2018)
Lacking nutrients—fruit peels	Supplementation of nitrogen waste	Deproteinized and demineralized prawn waste for chitin extraction	0.094–0.178 g chitin/g prawn waste	Conical flask, 100 ml working volume	*Bacillus subtilis* ATCC 6051 and *Lactobacillus plantarum* ATCC 14917 coculture	Aerobic	Many different peel wastes tested (red/white grape pomace, peels of apple, mango, potato, sweet potato, pineapple)	Tan et al. (2020)
Lacking nutrients—crude glycerol	Supplementation of nitrogen waste	Carotenoids	6.24 mg/l (approximately equal torulene and β-carotene)	5 l bioreactor, 3.5 l working volume	*Rhodotorula. gracilis* ATCC 10788	Aerobic	Potato wastewater, done at 20°C (more optimal than 28°C)	Kot et al. (2020)
Impurities—high potassium content in crude glycerol	Precipitate out potassium phosphate and sell	Lipids	19.5 g/l	1 l flask, 300 ml working volume	*Yarrowia lipolytica* SKY7	Aerobic	Economically viable process	Kumar et al. (2021)
Impurities—high salt concentration in waste cooking oil	Selection of halotolerant host strain	Rhamnolipid	1.1 g/l	150 ml flasks, 30 ml working volume	*Pseudomonas aeruginosa* M4	Aerobic	70 g/l salt in 25 g/l WCO as sole carbon source	Shi et al. (2020)
		Erythritol and lipase	22.1 g/l erythritol, 0.74 g erythritol/g WCO, 12.7 U/ml lipase	5 l bioreactor, 3 l working volume	*Yarrowia lipolytica* M53	Aerobic	80 g/l salt in 30 g/l WCO	Liu et al. (2017)
Substrate availability—lipid-rich wastes	Sonication to increase miscibility	Lipids	20.34 g/l, 0.51 ± 0.084 g lipid/g WCO	250 ml flask	*Cryptococcus curvatus* DSM-101032	Aerobic	Reported at 40 g/l initial sonicated WCO concentration	Patel & Matsakas (2019)
	Lipase hydrolyzation to increase free fatty acids	Lycopene	2.7 g/l	Two-stage fermentation, 250 ml flask to 1 l bioreactor	*Escherichia coli* FA03-PM	Aerobic	Oleic acid cofed with yeast extract and glucose had slightly higher lycopene titer (2.74 g/l) than hydrolyzed WCO cofed with the same substrates (2.65 g/l) and glucose alone (2.37 g/l)	Liu et al. (2020)
Toxicity—distillery wash	Dilution to reduce COD	Lipids	400 mg/l maximum	200 ml bubble column	*Chlorella vulgaris* CCAP211/19	Aerobic	Also removed color	Soleymani Robati et al. (2021)
Toxicity—hydrothermal liquefaction aqueous phase	Selection of tolerant host strain	Triacetic acid lactone (TAL)	21.6 g/l	3 l bioreactor	*Yarrowia lipolytica* Po1f base	Aerobic	Corn stover and HTL-AP supplemented (fed-batch)	Cordova et al. (2020)

## Carbohydrate-Rich Food Wastes

Despite many strategies to mitigate food waste (Gunders, 2012, p. 40), there are certain streams, like fruit peels and coffee grounds, for which waste is unavoidable. Food wastes more generically have potential uses as animal feed and composting, yet greater value can be extracted from these waste streams through the bioconversion of the fermentable sugars. In addition, food wastes often contain free amino nitrogen (FAN) or proteins, and sources of phosphate and other minerals necessary for microbial growth. As a result, carbohydrate-rich food waste streams can serve as low-cost microbial mediums with little need for supplementation. We highlight advances in the bioconversion of whey/acid whey and fruit peels as two exemplars of ongoing work in the field.

### Whey

Whey is a major by-product of the dairy industry that is formed when milk proteins like casein coagulate during yogurt production and cheesemaking (Chandrapala et al., 2015). Owing to high organic loads and large production volumes, whey is a major source of pollution—200 million tons are produced globally and approximately 50% is directly released into the environment (Barba, 2021; Valdez Castillo et al., 2020). Whey can be categorized into two groups: sweet whey that is derived from making rennet-type hard or semihard cheeses and acid whey that is generated during the production of soft cheeses and yogurts when coagulation is induced by a reduction in pH. Both forms of whey contain whey proteins, lipids, sugars (mainly lactose), lactic acid, minerals, and water soluble vitamins; however, acid whey has lower protein content, higher acidity, and higher mineral content (Menchik et al., 2019). As a result, acid whey is typically more difficult to process and valorize compared with sweet whey that has been successfully used in a variety of food products including sports drinks and infant formulas (Huang et al., 2019). Nevertheless, general production growth as well as popularity of products such as Greek yogurt have led to a 1–2% annual increase in acid whey production, thus increasing the need for efficient valorization strategies (Zerva et al., 2021).

Whey-based microbial growth media can be achieved through different levels of processing. Whole/crude whey is nonsterile, but has been successfully utilized as microbial media (Dessì et al., 2020; Israni et al., 2020; Mano et al., 2020). Whey powder, generated through spray drying, can be diluted in water to produce a medium with greater ability to control lactose concentration, albeit with higher production costs (Amaro et al., 2019; Zikmanis et al., 2020). Whey permeate is generated by removing the majority of proteins and solids, usually through heat treatment and ultrafiltration yielding a sterile homogenous solution that can also be spray dried into a whey powder. This final processing option yield a high carbon:nitrogen ratio, which is preferable for bioproduction of some products, such as polyhydroxyalkanoates (PHAs)—a class of biodegradable microbial polymers. In a recent study, *Bacillus megaterium*, was found to produce higher polyhydroxybutyrate (PHB) titers on whey permeate than on whole whey (Amaro et al., 2019; Das et al., 2018). Likewise, secondary cheese whey (scotta, generated from production of ricotta and cottage cheese), which has high lactose content but low protein content can be used for PHA production (Amaro et al., 2019).

To enable direct conversion of lactose, the primary sugar in whey, it is preferable to utilize organisms with β-galactosidase activity as this bypasses the need for additional pretreatment: hydrolysis via chemicals, enzymes, or *Lactobacilli* (Israni et al., 2020). For example, *Aureobasidium pullulans* (also known as *Aureobasidium namibiae*), a yeast-like fungus known to produce industrially relevant enzymes such as galactosidases (Prasongsuk et al., 2018), was recently studied for lipid production from whey powder medium (Wang, Ye, et al., 2021). Strain engineering can allow for the import of this enzymatic activity into cells that would otherwise be suitable organisms for this particular bioconversion. For example, many yeasts are unable to directly utilize lactose, however, they are often more suited for valorization of whey than bacteria due to their adaptation to low pH and tolerance to osmotic stress and nitrogen starvation (Valdez Castillo et al., 2020). In this regard, lactose consumption can be engineered easily (Beniwal et al., 2021; Brabetz et al., 1991; Jung & Lee, 2008; Mano et al., 2020). Among yeasts, those classified as Crabtree positive are less preferable when the desired product is not ethanol as the high lactose concentrations seen in whey will induce production of ethanol (Valdez Castillo et al., 2020). To demonstrate this point, recent engineering of hosts like *Yarrowia lipolytica* through heterologous expression of β-galactosidase and overexpression of native galactose utilization pathways can enable a platform for lipid production from acid whey (Mano et al., 2020). Mano et al. also used a *pex10* knockout to minimize hyphal formation and abolish peroxisomal β-oxidation, which improved lipid accumulation and enabled fermentation in concentrated acid whey. Furthermore, *Y. lipolytica* was able to maintain an optimal pH throughout this process for β-galactosidase activity by counterbalancing lactic-acid consumption with citric-acid generation. This feature, when combined with enhanced galactose metabolism could prevent bottlenecks in substrate uptake and enable full utilization of sugars in nonsterile unsupplemented acid whey (Mano et al., 2020). Looking forward, *Y. lipolytica* has native proteases and lipases (Zinjarde et al., 2014) which could allow for utilization of whey proteins and lipids in addition to the sugars.

Another organism capable of consuming the whey proteins and lipids is *B. megaterium* as described above. To demonstrate this, a β-galactosidase negative strain of this species was recently explored for PHA production from whole whey (Israni et al., 2020). Despite successes, several studies have found difficulty in achieving high biomass concentrations for organisms incapable of utilizing whey proteins without supplementing NH_4_^+^ (Conde-Báez et al., 2019; Shen et al., 2019). As a result, production of extracellular proteases and lipases may be a good criterion when selecting organisms for whey valorization.

In addition to hydrolytic activity, β-galactosidases can also catalyze transgalactosylation of lactose into galactooligosaccharides (GOS). Galactooligosaccharides are prebiotics with properties similar to the oligosaccharides found in human breast milk, commonly used in infant formulas (Fischer & Kleinschmidt, 2021). β-Galactosidases from *Aspergillus oryzae, Kluyveromyces lactis*, and *Bacillus circulans* are commonly used for commercial GOS production from lactose (Fischer & Kleinschmidt, 2021; Zerva et al., 2021). While these enzymes have been researched for GOS production from sweet and acid whey (Bolognesi et al., 2021; Fischer & Kleinschmidt, 2015; Mano et al., 2019; Simović et al., 2019), recent research has begun characterizing similar enzymes from other organisms such as *Thermothielavioides terrestris* (Zerva et al., 2021), *Cryptococcus laurentii* (Fischer & Kleinschmidt, 2021), and *Pantoea anthrophila* (Yañez-Ñeco et al., 2021). These enzymes have desirable properties such as greater thermostability, or better salt tolerance and low pH optima, which are especially useful for valorizing acid whey.

In addition to the products highlighted above, recent efforts in the microbial valorization of whey have included production of pigments (Mehri et al., 2021); enzymes such as β-galactosidases (Bentahar et al., 2019) and lipases (Knob et al., 2020); and polymers such as bacterial exopolysaccharides, bacterial cellulose, and chitosan (Kolesovs & Semjonovs, 2020; Kuppamuthu et al., 2020; Li et al., 2020). Collectively, these results demonstrate that both sweet and acid whey are feedstocks of interest for valorization.

### Fruit Peels

Like whey, fruit peels are another carbohydrate-rich waste stream of interest for biovalorization. However, the composition of fruit peels makes bioconversion more challenging than whey; and thus, laboratory studies have evaluated a variety of thermal, chemical, and mechanical pretreatment steps to enable enzymatic hydrolysis and microbial conversion. Ligninolytic organisms (especially some filamentous fungi and select ligninolytic bacteria (Xiong et al., 2020)) can directly ferment peel waste with only limited physical pretreatment. For example, a novel ligninolytic strain of *Alcaligenes faecalis* was recently employed for production of bioflocculants directly from citrus peel waste and could achieve a titer of 3.49 g/l under optimized conditions (Qi et al., 2020). Another study optimized watermelon peel waste as a cheap, new fungal medium and found that growth of several fungal species (*Aspergillus niger, Penicillium expansum, Fusarium oxysporum, Rhizopus oryzae, Lichtheimia corymbifera*) was comparable to or better than traditional potato dextrose (PD) with an additional advantage of being half the cost (Hasanin & Hashem, 2020). For nonligninolytic organisms, a traditional simultaneous saccharification and fermentation approach is possible. As an example, Yang et al. recently expressed an *A. niger* pectinase in *Pichia pastoris* to enable oligogalacturonide (OG) production directly from ground mandarin and orange peels (Yang et al., 2020). This was the first report of yeast engineered for OG production directly from citrus waste, and particularly substantial since OGs are traditionally produced using purified pectin through a multistep, costly process with negative environmental impacts.

Fruit peels vary in their content of lignin, thus impacting the type of pretreatment required and the composition of downstream microbial inhibitors such as phenolics, and organic acids released during enzymatic hydrolysis (Zeng et al., 2014). The issue of microbial inhibitors is repeatedly encountered across varied waste streams. In the case of fruit peels, these challenges are similar to the issues seen in lignocellulosic biomass hydrolysate. Specifically, the high cost of detoxifying hydrolysate of lignin-rich peel wastes disfavors the use of pretreatment leading to the search for more tolerant strains. For example, Siwina and Leesing identified a new strain of *Rhodotorula mucilaginosa* which achieved similar lipid yield and titer in raw and detoxified durian peel hydrolysate, despite the presence of inhibitors 5-HMF and acetic acid (Siwina & Leesing, 2021) (Table 1). A variety of peel wastes have been explored for biovalorization, including durian (Siwina & Leesing, 2021), pomegranate (Rayasam et al., 2020; Roukas & Kotzekidou, 2020), pineapple (Khan et al., 2021; Umesh et al., 2019), and watermelon. However, the majority of research has focused on citrus peels from industrial juice and marmalade production, as world production volumes are nearly 100 million tons annually (USDA Foreign Agricultural Service, n.d.; Khan et al., 2021), and 50–60 wt% of the actual fruit processed typically ends up as waste (peels, pulps, seeds) (Negro et al., 2016). Not surprisingly, citrus waste has recently been explored for the production of lipids (Carota et al., 2020), succinic acid (Patsalou et al., 2020), OGs (Yang et al., 2020), bioflocculants (Qi et al., 2020), and meso-galactaric acid (Protzko et al., 2018).

Fermenting citrus peels is likewise complicated by the presence of D-limonene and other plant essential oils that can inhibit microbial growth at very low concentrations (<1% vol/vol) (Satari & Karimi, 2018). At the same time, these essential oils are also highly valuable with D-limonene valued at $10 000/t for its use as a food preservative, flavoring, and eco-friendly cleaning agent (Negro et al., 2016). To this end, researchers have engineered organisms for its production as well as utilized limonene as a substrate for biotransformation to generate products such as limonene-1,2-diol and α-terpineol (Bier et al., 2017; Sales et al., 2018; Tai et al., 2016). Ongoing studies into the mechanism of limonene toxicity have identified organisms with natural tolerance to limonene (Lennartsson et al., 2012) and even some capable of utilizing limonene as a sole carbon source (Bier et al., 2011; Sales et al., 2018). Likewise, enhanced tolerance to limonene is possible via adaptive laboratory evolution, and rationally engineering genes identified via mutant screening, transporter libraries, and transcriptomics (Chubukov et al., 2015; Dunlop et al., 2011; Li et al., 2021; Ren et al., 2020). Nevertheless, owing to the potential high-valued stream, it is preferable to extract limonene and other essential oils through pretreatment with distillation, either before or after hydrolysis (Patsalou et al., 2020). Several examples of recent valorization schemes using distillation to remove plant essential oils from peel waste are highlighted in Table 1.

Also analogous to lignocellulose utilization, to derive the maximum value from peel wastes, microbial strains must be able to utilize the diverse carbon sources in the hydrolysate including pentoses (xylose, arabinose), hexoses (galactose, glucose, fructose, rhamnose), sucrose, and galacturonic acid (GalA) (Martins et al., 2020). Recent work has focused primarily on the utilization of GalA, an oxidized monosaccharide which composes over 70% of the pectin polymer, and is partially responsible for the highly acidic (pH 3.5) nature of citrus peel hydrolysates (Protzko et al., 2018). Abundant in citrus peels and other fruit wastes, pectin is a branched polysaccharide often used as a thickening/stabilizing agent in food products or as a matrix for edible films. This compound (like limonene described above) can be extracted from citrus peels to create another valorized by-product stream.

Both filamentous fungi and yeasts are well suited for acidic fermentation; however, GalA is not naturally utilized by the industrial workhorse Saccharomyces *cerevisiae* and many other yeasts of interest (*Y. lipolytica, Pichia stipitis, Kluyveromyces marxianus*) (Martins et al., 2020). An exception to this is the oleaginous yeast *Rhodosporidium toruloides* which could be a promising platform for biovalorization of citrus peel wastes. A recent genetic and enzymatic analysis showed that *R. toruloides* is capable of efficiently co-utilizing GalA and glucose or xylose with GalA rates 4 to 500 times higher than those reported for other well-known fungal species including *Neurospora crassa* and *A. niger* (Protzko et al., 2019). Nevertheless, there are challenges in consuming the array of sugars including a redox imbalance that can arise due to differing cofactor preferences among pathway enzymes. Catabolite repression (a consequence of glucose in the hydrolysate) is another difficulty encountered even among organisms capable of fermenting all monosaccharides in citrus peel hydrolysate, as demonstrated over 25 years ago with a recombinant strain of *Escherichia coli* (Grohmann et al., 1995).

Protzko et al. (2018) addressed both of these barriers through heterologous expression of the GalA specific transporter *GatA* in *S. cerevisiae* to enable co-uptake and utilization of glucose and GalA and also helped mediate redox issues using two strains with different GalA utilization pathways (Table 1). The first strain was engineered with the full fungal GalA catabolic pathway whereby GalA is converted into glycerol and pyruvate with simultaneous glucose catabolism in this strain regenerating NADH. A second strain was engineered for meso-galactaric acid (or mucic acid) production as an alternative to nitric-acid oxidation, a harsh, nonselective process which suffers from low yields and high costs (Liu et al., 2016). In this strain, meso-galactaric acid production from D-galacturonic acid is achieved by a single enzyme, uronate dehydrogenase (UDH) which uses NAD^+^ as a cofactor. Glucose co-utilization enhanced production, and further investigation indicated that this benefit arose from glucose acting as a redox substrate to regenerate NAD^+^, primarily through the formation of glycerol (Protzko et al., 2018). Jeong et al. expanded on this work to engineer a strain of *S. cerevisiae* capable of simultaneously consuming xylose, arabinose, and GalA, and found that the pentose sugars actually improved GalA utilization (Jeong et al., 2020). Intracellular metabolite analysis revealed that xylose and arabinose enhanced the final step of GalA conversion to glycerol—a promising finding since this conversion is catalyzed by an enzyme with reportedly low activities (Biz et al., 2016). These strategies could be applied to other host organisms to enhance production capacity from citrus peel waste.

Despite the advantages of peel hydrolysate with high sugar content, vitamins and other nutrients (especially nitrogen) are still necessary to enable robust microbial growth (Hasanin & Hashem, 2020). Toward this end, Patsalou et al. recently tested the impact of different nitrogen-source supplements on succinic acid production using *Actinobacillus succinogenes* alongside citrus peel waste and found that corn steep liquor gave the highest titers and nearly the same yields as yeast extract (Patsalou et al., 2020). Seafood waste can also provide a source of nitrogen and serve as a unique way to continue to utilize food wastes while overcoming nutrient limitations (Tan et al., 2020) (Table 1). These reports support continued research on the use of nitrogen-rich wastes in combination with fruit peel wastes to produce cost-effective microbial mediums for biomanufacturing.

### Lipid-Rich Wastes

The majority of lipid-rich waste streams are generated through the edible-oil industry as a result of processing—such as palm oil mill effluent (POME), or post consumption—such as waste cooking oil (WCO). Improper disposal of lipid-rich wastes can be toxic to ecosystems and form “fatbergs” that reduce sewer diameters or completely clog sewage pipes (Wallace et al., 2017). Traditionally, these wastes are thermochemically converted into biofuels or biodiesel, but their high free fatty acid and water content makes them largely unsuitable (Chen et al., 2016). However, these traits do not impede biological valorization. In fact, hydrolysis of lipids into free fatty acids is often the first step of microbial metabolism. Recently, a comprehensive review was published on microbial inhibitions and mitigation strategies of POME, known for its “acrid” qualities, dark color, and high amounts of biological oxygen demand (BOD) and chemical oxygen demand (COD) (Cheng et al., 2020). As a result, we highlight here other sources of lipid-rich waste and their biovalorization including WCO, fatty acid distillates (FADs), and waste animal fat. To improve bioavailability of these hydrophobic carbon sources, it is necessary to increase solubility in water, optimize cell culture or media including nutrient supplementation, and dilute the waste stream to optimize production or reduce inhibitor concentration.

### Waste Cooking/Frying Oil

Waste cooking oil is a post use product that can somewhat vary in composition as the act of cooking or frying oil (maintaining high temperatures for long times) and common kitchen contaminants such as water or salt can change properties such as color, solids, salinity, nutrient composition, and free fatty-acid content. The EPA estimates that over 14 lbs/person/year, or over 2.2 million tons of WCO was generated by restaurants nationwide (U.S. Environmental Protection Agency, 2014). The microbial valorization of WCO has been comprehensively reviewed recently (Lopes et al., 2020). Here we focus mainly on some highlights in the field such as the production of lipases; microbial oils/lipids; surfactants; or PHAs using a variety of bacterial, yeast, and filamentous fungal hosts. We also focus on a common valorization challenge associated with this stream, hydrophobic substrate availability.

The hydrophobic nature of WCO in an aqueous cell culture impacts substrate bioavailability. This problem has been approached chemically, mechanically, and biologically. Chemical and mechanical approaches involve pretreatments of WCO to increase surface area for cell contact. Biological approaches involve increasing lipase enzyme activity by adding exogenous lipase or optimizing process and media conditions. Surfactants as additives, or as value-added products themselves, have been studied as a chemical method to increase bioavailability and their effect on WCO valorization has likewise been reviewed recently (Lopes et al., 2020). It should be noted that there seems to be an optimal concentration of surfactant that is often strain, fermentation, or product specific (Lopes et al., 2020). However, these additives do come with a cost; and thus, mechanical methods such as sonication (the application of sound energy to a liquid, in this case to increase miscibility) have been investigated as an alternative. In this regard, sonicated WCO produced approximately 66% higher microbial lipids in *Cryptococcus curvatus* DSM-101032 compared to untreated WCO (Patel & Matsakas, 2019), likely owing to a reduced lipid droplet size and increased cellular uptake (Table 1). Waste cooking oil fermentation with lipase treatment along with glycerol produced 2.7 g/l lycopene from *Escherichia coli* (Liu et al., 2020) (Table 1). Recently, the immobilization of *Thermomyces lanuginosus* lipase on the surface of *Y. lipolytica* Po1h not only increased specific lipase activity, but also improved bioconversion of WCO into fatty-acid methyl esters, due to the forced proximity of lipase and the cell (Qiao et al., 2018).

The composition of WCO, especially inhibitors, can impact valorization. It is thus important to select or acclimate a proper host strain for these conditions. For example, a main inhibitor in WCO is table salt, so halotolerant organisms are appropriate hosts for high-salt WCO valorization. Recently, *Pseudomonas aeruginosa* M4 was shown to tolerate up to 70 g/l of salt, and produce rhamnolipid surfactant up to 1.1 g/l (Shi et al., 2020). High salt content is also an asset in some fermentations (Table 1). For example, it has been identified as crucial for erythrol production in the yeast *Y. lipolytica* M53 where 22.1 g/l of erythritol was produced along with lipases from WCO at 80 g/l salt (Liu et al., 2017). Another research group, hypothesizing that native cultures may have evolved superior growth on WCO, isolated *Bacillus cereus* MPTDC from sewage, which was able to produce 3.7 g/l of PHB with media optimization (Sindhu et al., 2020). Similarly, 11 g/l of rhamnolipids were produced by *Pseudomonas aeruginosa* MTCC7815 at an optimal pH of 10, similar to natural sewage conditions (Sharma et al., 2019). Additionally, an indigenous strain of *Pseudomonas aeruginosa* NJ2, isolated from frying-oil condensate, was able to produce 4.3 g/l rhamnolipids (Pathania & Jana, 2020a). With further optimization of growth conditions, production titers were increased to 6.3 g/l rhamnolipids (Pathania & Jana, 2020b).

Recent advances in metabolic engineering have been most successful when simultaneously considering host organism and metabolism. For example, by diverting metabolic flux toward the β-oxidation pathway via deletion of the *tctA* gene (involved in preferred carbon-source transport), medium chain poly(3-hydroxyalkanoates) production increased nearly twofold in *Pseudomonas putida* KT2440 to 1.9 g/l (Borrero-de Acuña et al., 2019). Another group found that by increasing oxidative stress in *Blakeslea trispora* cultures, β-carotene, γ-carotene, and lycopene were produced at a total titer of 980 mg/l (composed of 71%, 26%, and 3%, respectively) (Nanou et al., 2017). *Y. lipolytica* base strain Po1g was also metabolically engineered to produce 2.5 mg/l and 2.7 mg/l of D- and L-limonene, respectively (Pang et al., 2019). The long-chain monounsaturated fatty-acid erucic acid was also produced at titers of 887 mg/l from metabolically engineered *Y. lipolytica* Po1d (Gajdoš et al., 2020). Continued efforts on microbial valorization of WCO should focus on proper host-strain selection in parallel with increasing bioavailability.

### Fatty Acid Distillates

Fatty acid distillates are a major by-product formed in the processing of vegetable oils, especially those of soybean (SFAD) or palm (PFAD). For example, approximately 600 000 tons of PFAD are produced annually amounting to 3.6% of all crude palm oil (Radzuan et al., 2018). Fatty acid distillates are mainly composed of free fatty acids (greater than 85%) with the remaining content being TAG, sterols, tocopherols, or volatile compounds, and have traditional uses in the production of soap or animal feeds (Aguieiras et al., 2019). Although extensive research has been done on the chemical catalysis of FAD for biofuels production, little literature is available on microbial valorization with the exception of rhamnolipid surfactant production and specifically, palm FAD.

In 2017, it was reported that researchers used *P. aeruginosa* PAO1 to produce 0.4 g/l rhamnolipid from palm FAD and complex media (Radzuan et al., 2017). By surveying PFAD concentrations of 20, 50, and 100 g/l, it was found that 100 g/l of PFAD had the highest rhamnolipid titers, but only by very slightly (0.38, 0.39, and 0.43 g/l rhamnolipid with 20, 50, and 100 g/l PFAD, respectively). Taking these results, another group hypothesized that this limitation was due to poor bioavailability. To test this hypothesis, they slowly added heated, liquefied PFAD at a final concentration of 10 g/l into their media to increase surface area. This produced a maximum of 1.8 g/l rhamnolipid in *Pseudomonas* sp. LM19 (Nurfarahin et al., 2019). By further optimization of nutrient concentrations and use of minimal media, Radzuan et al. increased their titer almost ninefold to obtain 3.4 g/l rhamnolipid on 10 g/l PFAD as the sole carbon source (Radzuan et al., 2018). Beyond rhamnolipids, the production of enzymes such as lipases from palm oil deodorizer distillate using wild-type *Y. lipolytica* IMUFRJ 50682 has been demonstrated although lipase production (2.3 U/ml) was about twofold lower than that produced from residual frying oil (Fraga et al., 2021).

The exact composition of FADs may influence overall processes. There is some emergent literature on microbial valorization of other streams beyond PFAD. For example, a variety of strains were screened for PHA production using fatty acids as feedstocks containing wastes including olive oil distillate (OOD). The highest titer measured was 5.5 g/l PHA in strain *Cupriavidus necator* DSM 428, interestingly higher than the PHA titer of the same strain grown on WCO (4.6 g/l) (Cruz et al., 2016). It was hypothesized that the presence of sterols and tocopherols may have actually enhanced growth, although this was not further investigated. Another group used sunflower acid oil to produce rhamnolipids at 4.9 g/l in *P. aeruginosa* MTCC 2453, outperforming reported literature production using WCO, glycerol residue, and olive mill waste at the time (Jadhav et al., 2019). As FADs are an emerging waste stream for microbial valorization, future work should first focus on media and culture optimization in addition to identifying and neutralizing potential microbial growth inhibitors or promoters.

### Waste Animal Fat

Waste animal fat is a particularly large waste stream in the livestock industry and this lipid-rich waste stream has a higher percentage of saturated fats (almost 40% total) compared with other streams discussed above (Toldrá-Reig et al., 2020). As a result, aqueous fermentations are challenging, and thus the majority of existing literature focuses on solid-state fermentation that are limited by long fermentation times along with heat and/or mass transfer limitations (Krishania et al., 2018). Recently, a group investigated methods for increasing the solubility of waste animal fat for aqueous cell culture using lipase pretreatments, emulsification, and hydrolysis (Szotkowski et al., 2019). By testing across different red yeasts (*Cystofilobasidium macerans* CCY 10–1–2, *Rhodotorula glutinis* CCY 20–2–26, *Rhodotorula mucilaginosa* CCY 19–4–6, and *Sporobolomyces pararoseus* CCY 19–9–6), they discovered that reducing the carbon/nitrogen (C/N) ratio to 13 obtained the highest titers of desired products including carotenoids, ergosterol, and ubiquinone. Their highest reported titer of carotenoids, 24.8 mg/l, was produced in *Sporobolomyces pararoseus* CCY 19–9–6 on emulsified fat, an approximately fourfold improvement over crude fat. This strain also featured the highest ubiquinone titer, 11.3 mg/l, on hydrolyzed fat—an over twofold increase from crude fat. *Rhodotorula mucilaginosa* CCY 19–4–6 had the highest ergosterol titer on glycerol of 0.68 mg/l, only 1.1-fold higher than crude fat. Clearly, the hydrolysis or emulsification of waste animal fat increases its valorization potential, and supplementation of glycerol or other carbon or nutrient sources should be investigated in future work.

The optimization of cell-culture parameters has also been explored. For example, the effect of initial pH, Arabic gum surfactant concentration, waste fat concentration, and oxygen transfer rate (OTR) in *Y. lipolytica* W29 on lipid accumulation from pork lard was investigated through Taguchi experimental design (Lopes et al., 2018). It was found that OTR was the most influential on lipid accumulation, and the lipids produced by *Yarrowia* had a higher unsaturated:saturated fatty-acid ratio, indicating higher suitability for animal feed. It has also been shown recently that salt addition increased the amount of volatile fatty acids produced in the AD of waste animal fat, more so than waste vegetable fat (Liu & Jiang, 2020). This would be an interesting avenue to explore for increasing solubility of the saturated fatty acids in animal fats for future work, which currently appears to be the largest challenge associated with this waste stream.

## Other Industrial Wastes

Outside of food and food processing waste, there are a significant number of other industrially related waste streams that can be valorized. While increased regulations, smarter process development, and a growing interest in green chemistry have helped reduce industrial waste, ecologically toxic waste is still produced by many major manufacturing industries. Treatment of these wastes through valorization is not only crucial to the development of a circular economy, but also highly incentivized due to heightened disposal costs. Here, we highlight representative examples of the valorization of other nonconventional and underutilized wastes in industry including crude glycerol, hydrothermal liquefaction processes, crude oil, and distillery wash.

### Crude Glycerol

Crude glycerol is generated as a by-product from the transesterification of triglycerides into biodiesel with approximately 10 kg of crude glycerol generated for every 100 kg of biofuels produced. Annually, this amounts to over 2 billion liters of crude glycerol generated globally (Rodrigues et al., 2020). Although pure or refined glycerol has a variety of uses in industry, crude glycerol is not as suitable for traditional markets or chemical conversions due to a variety of impurities, such as salts, soaps, triglycerides, catalysts, free fatty acids, or methanol (Haron et al., 2018). Due to variations in these impurity compositions, it is challenging to have a “one size fits all” approach for the valorization of crude glycerol. Efforts in the field have demonstrated strong potential from this feedstock, including the valorization (by *E. coli*) to produce ethanol, 1,2-propanediol, and L-lactate (Clomburg & Gonzalez, 2011; Mazumdar et al., 2013; Shams Yazdani & Gonzalez, 2008). The valorization of crude glycerol for the chemical and biological production of fuels and chemicals such as 1,3-propanediol, lipids, hydrogen, succinic acid, and citric acid has been reviewed recently (Kosamia et al., 2020) as a complement to the analysis provided here.

Recent literature has shown that oftentimes, the impurities in crude glycerol may be an asset in microbial valorization. For example, the production of cyclopropanated fatty acids from crude glycerol in metabolically engineered *Y. lipolytica* Po1d showed a 2.4-fold increase in titer (7.5 g/l) relative to pure glycerol (Imatoukene et al., 2020). However, the specific effect of potential impurities that may explain this was not studied. In another study, crude glycerol was a better substrate for *Rhizomucor miehei* lipase production in *P. pastoris* GS115 than pristine glycerol and was found to be enhanced by higher concentrations of Na^+^, Ca^2+^, and grease impurities although inhibited by higher Fe^3+^ (Tian et al., 2021). Moreover, for this specific fermentation, the presence of methanol was an asset as methanol-based promoter regulation is common in *P. pastoris.* In another study investigating methanol impurities in glucose, a group found that *R. toruloides* ATCC 10788 was inhibited by methanol impurities in crude glycerol as low as 0.5% (wt/vol), but this impact was masked by the positive impact of other impurities at higher concentrations of crude glycerol leading to an overall net increase in lipid biomass (Uprety et al., 2017). By optimizing waste glycerol concentration as well as other relevant culture/media parameters, docosahexaenoic acid was produced at 17.3 g/l in *Schizochytrium* sp. (Kujawska et al., 2021).

However, not all contaminants in crude glycerol are beneficial as suggested in literature. For example, high levels of sodium, sulfur, and potassium contaminants have each been shown to inhibit growth and citric-acid production from glucose in *Y. lipolytica* SKY7 (Kumar, Yellapu, Yan, et al., 2020). To address high potassium levels, the group proposed pretreating crude glycerol with phosphoric acid, to precipitate out and sell the resulting potassium phosphate (Table 1). Following this treatment, cells produced lipid contents up to 19.5 g/l that were suitable for biodiesel (Kumar, Yellapu, Tyagi, et al., 2021). Contrary to these reports, not all waste glycerol streams are easy to valorize. While a bacterial consortium isolated from activated sludge and improved via serial subcultures can produce up to 27.8 g/l of 1,3-propanediol and 14.7 g/l lactic acid from crude glycerol derived from hydrolyzed vegetable oils, this same consortia failed to produce 1,3-propanediol and only made 2.9 g/l lactic acid when grown on WCO-derived glycerol despite complete carbon utilization (Wang et al., 2019). It is also possible that crude glycerol has no effect on fermentations. A recent study found that the production of microbial lipids and carotenoids from waste frying oil (WFO) derived glycerol in *Rhodotorula* sp. had no inhibitory effects relative to pristine glycerol (Pino-Maureira et al., 2021). It is likely that differences in the waste stream, culture conditions, microbial host, or some combination are causing these discrepancies and warrant further investigation.

Many wastes containing glycerol lack nitrogen sources, an essential nutrient in cell culture. One way to source this nitrogen is to ferment with a nitrogen-rich waste source in addition to glycerol. Nitrogen-rich crustacean waste was added to a *Y. lipolytica* SM7 crude glycerol fermentation to increase lipase production from 25 U/ml to 38 U/ml (Magdouli et al., 2017). Nitrogen-rich potato wastewater supplementation in a crude glycerol fermentation of *Rhodotorula gracilis* ATCC 10788 produced carotenoids at 6.24 mg/l (Kot et al., 2020). Other supplementations have also been explored. For example, *Paenibacillus polymyxa* PM 3605 was shown to produce approximately 14.5 g/l of 2,3-butanediol from 50 g/l crude glycerol, and 19 g/l when supplemented with 10 g/l of sugarcane molasses (Tinôco et al., 2021). Future work in the valorization of crude glycerol should involve continued characterization of potential impurities and deficiencies that may both increase and decrease titer, and a careful consideration of how the host strain and the desired product could impact microbial valorization.

### Hydrothermal Liquefaction Aqueous Phase

Hydrothermal liquefaction aqueous phase (HTL-AP) is the aqueous phase of waste generated from hydrothermal liquefaction processing of biomass to generate biofuel or biocrude at high temperatures and pressures (Skaggs et al., 2018). This waste contains toxic organic compounds such as alcohols, acids, ketones, aromatic compounds, and N-heterocyclic compounds and possesses a high COD, yet currently has no industrial use (Seyedi et al., 2020). While its toxicity poses a high challenge for valorization, reduction of treatment costs would be of enormous benefit. Anaerobic digestion is currently the main method of HTL-AP bioremediation, and has been reviewed recently (Seyedi et al., 2020).

Microbial valorization will require either reducing toxicity through pretreatments (such as air stripping and solvent extraction) or improving tolerance by selecting a host strain more tolerant to HTL-AP. The latter was demonstrated using a metabolically engineered *Y. lipolytica* where HTL-AP was valorized into either triacetic acid lactone (TAL) or itaconic acid (Cordova et al., 2020) (Table 1). In defined media, the addition of 10% HTL-AP actually improved both TAL and itaconic acid titer. HTL-AP at 20% was supplemented with mixed sugar hydrolysate to produce 21.6 g/l of TAL at the bioreactor scale. This study is one of few demonstrations of HTL-AP microbial valorization potential as the majority of literature is focused on improving microbial detoxification or AD strategies (Seyedi et al., 2020; Wang et al., 2021). Future work to expand the utility of this waste stream should involve further host strain engineering especially in the area of tolerance.

### Crude Oil

Crude oil is a complex mixture of hydrocarbons, n-paraffin, and aromatics along with high levels of sulfur, nitrogen, and asphaltene especially in heavier or sour crudes (Marafi et al., 2019) (Li, Zhou, et al., 2021). Often, spillages may contaminate surrounding soils and ecosystems, or may otherwise be unusable for production; and thus, much work has focused on the bioremediation of crude oil. As for using crude oil as a direct carbon source, efforts have focused mainly on the production of surfactants (as reviewed recently [Dhanya, 2021]) in addition to rhamnolipids to remediate crude oil specifically (Gaur & Manickam, 2021). Beyond these approaches, new and interesting microbes have been discovered near oil-contaminated areas with Gram-negative bacteria (such as *Pseudomonas* sp. and *Ochrobactrum* sp.) typically reported as being better at degrading oil than Gram positive (Wu et al., 2018). This may be due to a higher reported tolerance to saturated and aromatic hydrocarbons relative to Gram-positive bacteria (Marilena Lăzăroaie, 2010). Recently, *Pseudomonas aeruginosa* WD23 isolated from petroleum refinery effluent was shown to degrade up to 27% crude oil in seawater with minimal supplementation (Goveas et al., 2020). However, Gram-positive bacteria may also have valorization potential. As an example, Gram-positive *Staphylococcus capitis* strain SH6 has been shown to degrade crude oil (either motor or diesel oils) and additionally produce native biosurfactant (Chebbi et al., 2018). Recently, a bacterial exopolysaccharide emulsifier was produced from natively isolated *Geobacillus stearothermophilus* DG1 that not only solubilized crude oil, increasing its bioavailability, but was also shown to degrade the resulting alkanes and alkyl hexanes (Li, Zhou, et al., 2021). Clearly, future crude oil valorization should focus on selection of the proper host strain, and the target product, which initially will be microbial lipids for biofuels or a surfactant. As growth on this oil as a sole carbon source is still emerging, microbial acclimation through techniques such as adaptive laboratory evolution may be an interesting microbial valorization method.

### Distillery Wash

Ethanol fermentation generates approximately 12 l of wastewater per liter of alcohol in a product known as distillery wash or distillery stillage (Mikucka & Zielińska, 2020). This waste stream has high BOD, COD, is often dark in color, and may contain sugars, solids, sulfates, phosphates, and metals (Chowdhary et al., 2017). The nonmicrobial valorization of distillery stillage has been reviewed recently, focusing mainly on the chemical extraction of valuable components such as volatile fatty acids, polyphenols, and polysaccharides (Mikucka & Zielińska, 2020). However, there are still challenges associated with solvent extraction, including selectivity, environmental friendliness, and toxicity. Thus, microbial valorization of this stream is of high interest.

The oleaginous yeast *Metschnikowia pulcherrima* was grown on distillery wash and could produce fatty-acid methyl esters with a titer of 1.4 g/l after optimization of culture time, pH, and temperature (Anbarasan et al., 2018). Recently, it has been shown that the algae *Chlorella vulgaris* CCAP 211/19 can both remove colorants in this waste stream and produce oleaginous biomass (Soleymani Robati et al., 2021), which may be of use for biofuels production. An approximately 30-fold dilution of the distillery wash was used to reduce the COD to 2 g/l, with the purpose of reducing its inhibitory effects (Table 1). Potassium may be an important (and sometimes limiting) factor in the production of microbial lipids from this waste stream. For example, post-methanated distillery wash was shown to produce up to 10 g/l citric acid in *Aspergillus fumigatu*s PN12 MH791044 with the additional optimization of potassium phosphate (Aghera & Bhatt, 2019). A culture of *Y. lipolytica* MUCL30108 was shown to produce microbial lipids suitable for biofuel production using distillery spent wash pretreated with *A. niger* cellular extracts (Hoarau et al., 2020). Future research into physical and chemical pretreatment methods as well as supplementations may further improve microbial valorization potential for this waste stream.

## Conclusion

Currently, the valorization of nonconventional and underutilized waste streams has not reached its full potential. However, these approaches are critical to achieve a sustainable, circular economy. The production of high-value products may mitigate the economic challenges and outcompete established, lower-value methods such as composting, animal feed, or AD. Although life-cycle assessments and systemic analyses have been conducted on many waste streams, to the best of the author's knowledge at the time of publication, commercialization of microbial valorization (within the scope of this review) has not been achieved. However, some cases have demonstrated titers at or close to industrial scale (Table 1) and the commercialization of similar processes in the near future has great promise and potential.

The valorization of nonconventional and underutilized wastes involves many challenges including nitrogen content, impurities, toxicity, and low productivity, as well as bioavailability. Further research should focus on identifying and developing robust host strains, especially for streams with high impurities and toxicity. Strains newly isolated from waste sources often possess the required tolerance and catabolic traits but may lack the advantages of well-studied hosts such as knowledge of relevant metabolic pathways, ease of cloning, established culture techniques, and safe/nonpathogenic status. If the mechanism of tolerance is easily understood, porting these traits into well-studied hosts is preferable. Well-studied hosts may also be improved through adaptive evolution. In addition, future work should focus on optimizing pretreatments; media formulation (including mixing waste streams); and culturing techniques to address bioavailability, nutrient deficiency, and toxicity in a dynamic method for the most optimal microbial valorization.

## Data Availability

Data sharing is not applicable to this article as no new data were created or analyzed in this review.
